# The Variability of Lumbar Sequential Motion Patterns: Observational Study

**DOI:** 10.2196/41906

**Published:** 2023-06-20

**Authors:** Inge Caelers, Toon Boselie, Wouter van Hemert, Kim Rijkers, Rob De Bie, Henk van Santbrink

**Affiliations:** 1 Department of Neurosurgery, Zuyderland Medical Center Heerlen Netherlands; 2 Care and Public Health Research Institute Maastricht University Maastricht Netherlands; 3 Department of Neurosurgery, Maastricht University Medical Center + Maastricht Netherlands; 4 Department of Epidemiology, Maastricht University Maastricht Netherlands

**Keywords:** lumbar spine, cinematographic recordings, sequence, motion pattern, flexion, extension, rotation, physiological, musculoskeletal, motion, spine, upper lumbar, observational study, physiological motion

## Abstract

**Background:**

Physiological motion of the lumbar spine is a topic of interest for musculoskeletal health care professionals since abnormal motion is believed to be related to lumbar complaints. Many researchers have described ranges of motion for the lumbar spine, but only few have mentioned specific motion patterns of each individual segment during flexion and extension, mostly comprising the sequence of segmental initiation in sagittal rotation. However, an adequate definition of physiological motion is still lacking. For the lower cervical spine, a consistent pattern of segmental contributions in a flexion-extension movement in young healthy individuals was described, resulting in a definition of physiological motion of the cervical spine.

**Objective:**

This study aimed to define the lumbar spines’ physiological motion pattern by determining the sequence of segmental contribution in sagittal rotation of each vertebra during maximum flexion and extension in healthy male participants.

**Methods:**

Cinematographic recordings were performed twice in 11 healthy male participants, aged 18-25 years, without a history of spine problems, with a 2-week interval (time point T1 and T2). Image recognition software was used to identify specific patterns in the sequence of segmental contributions per individual by plotting segmental rotation of each individual segment against the cumulative rotation of segments L1 to S1. Intraindividual variability was determined by testing T1 against T2. Intraclass correlation coefficients were tested by reevaluation of 30 intervertebral sequences by a second researcher.

**Results:**

No consistent pattern was found when studying the graphs of the cinematographic recordings during flexion. A much more consistent pattern was found during extension, especially in the last phase. It consisted of a peak in rotation in L3L4, followed by a peak in L2L3, and finally, in L1L2. This pattern was present in 71% (15/21) of all recordings; 64% (7/11) of the participants had a consistent pattern at both time points. Sequence of segmental contribution was less consistent in the lumbar spine than the cervical spine, possibly caused by differences in facet orientation, intervertebral discs, overprojection of the pelvis, and muscle recruitment.

**Conclusions:**

In 64% (7/11) of the recordings, a consistent motion pattern was found in the upper lumbar spine during the last phase of extension in asymptomatic young male participants. Physiological motion of the lumbar spine is a broad concept, influenced by multiple factors, which cannot be captured in a firm definition yet.

**Trial Registration:**

ClinicalTrials.gov NCT03737227; https://clinicaltrials.gov/ct2/show/NCT03737227

**International Registered Report Identifier (IRRID):**

RR2-10.2196/14741

## Introduction

Physiological motion of the lumbar spine is of interest for musculoskeletal health care professionals. Motion of the lumbar spine is dependent on multiple structures, for example facet joint orientation, spinal-pelvic relations, intervertebral disc loading, and muscle recruitment. Although the concept of physiological motion is used in many instances, a proper definition is still lacking. Over the last 90 years, several attempts to define physiological motion have been made. In 1931, Dittmar et al [1] were the first to use sagittal radiographs to analyze the normal range of flexion and extension for the lumbar spine. Subsequently, more motion research followed using other techniques including computed tomography and magnetic resonance–based 3D imaging [2-4]. Based on these data, segmental ranges of motion with a high intra, and interindividual variability were described [5,6]. For this reason, researchers started to investigate sequences, like sequence of segmental initiation of motion. Studies that report sequence of segmental initiation of motion in flexion and extension also showed variable results. The lack of consistent segmental ranges of motion or sequence hampers the definition of physiological motion of the lumbar spine [7-17].

Our research group described a consistent sequence of segmental contribution in the lower cervical spine during extension using sagittal cinematographic recordings [18]. This research was used to create a definition of physiological motion in young healthy individuals without spinal complaints. To our knowledge, similar analysis of the sequence of segmental contribution for the lumbar spine has not been carried out previously.

This study aimed to analyze the sequence of segmental contribution of L1 to S1 in sagittal rotation during flexion and extension in individual participants. A consistent pattern of segmental contribution in asymptomatic participants could be seen as a definition of psychological motion. In the future, this pattern could be used to investigate potential abnormal motion in lumbar conditions. It might be possible to better diagnose instability and the impact of it on lumbar spine motion. Furthermore, we can determine if differences in motion lead to back pain and can be resolved by physiotherapy.

## Methods

### Ethics Approval

The study was approved by the Medical Research Ethics Committee of Zuyderland Hospital and Zuyd University of Applied Sciences, the Netherlands (METCZ20180094).

### Participant Inclusion

The study protocol was published [19]. After approval, this study included men, aged between 18 and 25 years, with a BMI <25 kg/m^2^, with no medical history of spine problems, and able to perform maximum lumbar flexion and extension without complaints. No medical history of spine problems was defined as no visits to a doctor or physical therapist for spine complaints, no former spine surgery, total scores of Oswestry Disability Index and Visual Analogue Scale for back pain of zero, and a Kellgrens’ classification of 0-1 in levels L4L5 and L5S1 on cinematographic recordings evaluated by 2 spine surgeons (TB, HvS, and WvH) [20-22]. Female participants were excluded to protect their ovaries from direct radiation exposure. Potential participants were excluded if x-rays of the abdomen, pelvis, hip, lumbar, or sacral spine were taken in the previous year or in cases of active spinal infection, immature bone, lumbar tumor, previous lumbar radiotherapy, congenital lumbar spine abnormality, or planned pregnancy of the participants’ partner in the coming year. Sample size, based on previous studies, was set on 11 participants [13,14,23].

Informed consent was acquired from all participants. Radiological data were stored along with the number of participants and recordings. Handling of personal data will comply with the guidelines of the Dutch Personal Data Protection Act.

### Study Procedures

**Figure 1:**
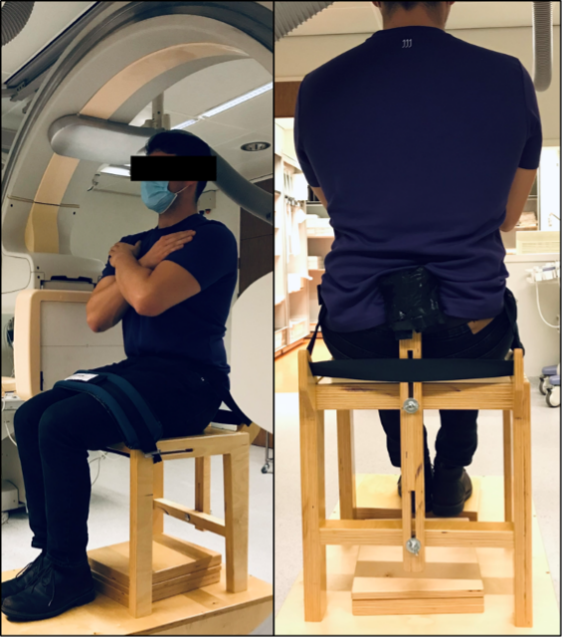
Customized wooden chair with 3-point fixation of the pelvis. The 3-point fixation is located on the anterior superior iliac spine, posterior inferior iliac spine, and the upper legs.

Flexion and extension cinematographic recordings were acquired twice for each participant during afternoons and evenings. An interval of 2 weeks was maintained to determine reproducibility and consistency of the sequence between 2 time points (T1 and T2) [18,24]. Cinematographic recordings were made from a lateral perspective to obtain sagittal images, using the Philips Allura Xper FD20 x-ray system. The following settings were used: frames of 1024×1024 pixels, 7.5 frames per second, tube voltage of 75-90 kV, filter of 0.9 mm copper + 1 mm aluminum, and a detector distance of 48 cm. The total radiation dose for participants was categorized in category 2A, using the Neurocritical Care Society guidelines on risks of radiation dose (0.1-1.0 mSv) [25]. During cinematographic recordings, participants were seated in a customized wooden chair, designed to keep the pelvis in a fixed position (Figure 1). A 3-point fixation was located on the anterior superior iliac spine, posterior inferior iliac spine, and the upper legs, which could be adjusted to the participants’ physique. Participants were asked to remove clothes that could disturb the cinematographic recordings. From a neutral seating position with the knees in 90 degrees flexion, participants were asked to perform maximum extension, followed by maximum flexion, and then a return to maximum extension in 14 seconds, using a metronome. Maximum flexion and extension was determined as the maximum achievable position of the participant and practiced before the final cinematographic recordings. During the active motion task, arms were crossed in front of the chest (Figure 1). This duration was chosen based on the pulse frequency of the image technique (7.5 pulses per second) and the number of necessary images (104 images) for image recognition.

### Radiological Data Processing and Analysis

For this research, we have previously developed custom software that uses image recognition algorithms to track vertebrae during flexion and extension [26]. The software follows bony structures within user-defined template areas throughout all frames, using a best-fit principle to match normalized gradient field images. To define these template areas, the user draws polygons around all vertebrae on the median frame of the recording [26]. After the software has completed tracking these structures, they can be manually evaluated. Corrections can be made if necessary. Finally, graphs are made for both flexion and extension cinematographic recordings for each individual participant to identify specific patterns in the sequence of segmental contributions. Segmental rotation of each individual segment (L1 to S1) between each pair of successive frames was plotted against the cumulative rotation in segments L1 to S1 together. A more detailed description of the image recognition software can be found in a previously published study [26]. Analyses were first performed for T1 and tested against T2. Time spent on radiological data processing and analysis was 2 to 3 days per cinematographic recording. Analyses were performed by researcher IC, with reevaluation of 30 intervertebral sequences by a second researcher (TB) to determine reproducibility, using a two-way mixed intraclass correlation coefficient (ICC). An ICC above 0.60 was considered adequate. A consistent motion pattern was defined as a similar pattern shown in at least 80% (8/10) of the cinematographic recordings in 2 time points. This was comparable with the results of the cervical spine [18].

## Results

**Figure 2:**
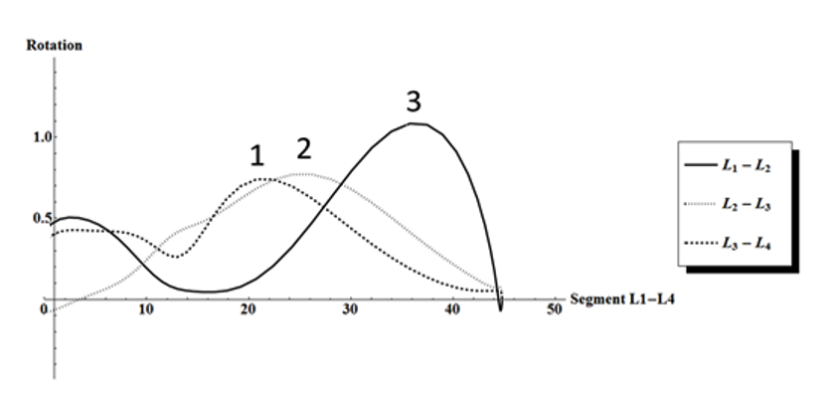
Sagittal rotation in segments in the upper lumbar spine (segments L1 to L4) during extension of the lumbar spine in healthy young male participants (P2-T1). On the y-axis, the rotation is shown in degrees between successive frames. On the x-axis, cumulative degrees of extension in block L1 to L4 are shown. Peaks of the graphs per segments (L1L2, L2L3, and L3L4) depict maximum contribution of the segment in a specific phase of the extension. At the last phase of the extension, the L3L4 peak was followed by an L2L3 peak and finally the L1L2 peak. Each series of values undergoes smoothing by means of a low-pass Gaussian digital filter.

A total of 11 participants were recruited and included, all undergoing 2 cinematographic recordings. This resulted in a total of 22 recordings, of which 1 (P1-recording 1) was excluded from analyses, since L1 could not be followed in the field of view. No consistent pattern was found when studying the graphs of the cinematographic recordings during flexion (Multimedia Appendix 1). During extension, segments L4L5 and L5S1 showed an inconsistent pattern (Multimedia Appendix 2). Leaving L4L5 and L5S1 out of the analyses, a much more consistent pattern on the sequence of segmental contribution was found, especially in the last phase of the extension motion. It consisted of a peak in rotation in L3L4, followed by a peak in L2L3, and finally, in L1L2 (Figure 2; Multimedia Appendix 3). Only the sequence of the peaks was important, not the height of the peaks itself, since a peak represents the largest contribution of a specific segment at a specific point in the total motion despite the height. As discussed in the study of Boselie et al [18], peaks with a rotation lower than 0.3 were deemed to fall within the measurement error and were not taken into consideration. In total, 71% (15/21) of extension graphs showed the abovementioned sequence, which represents 80% (8/10) at T1 and 64% (7/11) at T2 (Multimedia Appendix 3). At both time points, P5 and P7 did not show a consistent motion sequence with different motion patterns at each time point. P9 only showed a consistent motion sequence in T1. ICC was determined for each segment in 5 cinematographic recordings (Table 1).

**Table 1:** Intraclass correlation coefficients (ICC) per segment of 5 randomly chosen cinematographic recordings. An ICC below 0.60 is determined as inadequate and indicated in italics.

Segments	Cinematographic recordings					
	2-1	3-2	4-1	8-1	11-2	Mean
L1L2	0.601	0.858	*0.389*	0.662	*0.550*	0.612
L2L3	0.799	0.615	0.808	0.662	0.722	0.721
L3L4	*0.577*	0.819	0.886	0.932	0.694	0.782
L4L5	0.691	*0.437*	0.876	0.917	*0.553*	0.695
L5S1	0.763	*0.258*	0.750	0.902	*0.268*	*0.588*

## Discussion

### Principal Findings

The aim of this study was to ascertain the sequence of segmental contribution and to possibly understand physiological motion in sagittal rotation during maximum flexion and extension of the lumbar spine in asymptomatic male participants. Results showed a consistent pattern in 71% (15/21) of the recordings during the last phase of the extension with a peak in rotation in L3L4, followed by a peak in L2L3, and finally, in L1L2. However, this pattern was consistent in only 64% (7/11) of the recordings over the 2 time points.

Previous studies have used different imaging techniques to describe the range of motion and the sequence of initiation of motion of individual segments during flexion and extension of the lumbar spine. Dvorak et al [16], Pearcy et al [15], and Staub et al [11] described range of motion in rotation during maximal passive flexion and extension of each level. Since range of motion differed between studies and resulted in a high inter, and intraindividual variability, a more consistent method to define physiological motion was pursued. Initiation of motion was described by several previous studies. Because of limitations (eg, reporting pooled data instead of individual sequences, limited range of motion, analyses of part of the lumbar spine, and describing intervertebral rotation at specific time points or specific ranges of motion instead of between successive frames), results differ between studies with high inter and intraindividual variability [7-10,13,14,17].

In a cervical spine study [18], a more consistent sequence of segmental contribution during the end of the extension, namely in 80% of the participants in T1 and 90% in T2, was found using the same measurement method and setup as this study. The reliability, sensitivity, and specificity of this measurement method showed high scores, with a reliability, determined in Fleiss Kappa, of 0.80-0.84, average sensitivity of 90%, and average specificity of 85% [18]. We believe that these findings show that this method is accurate and reproducible to determine the sequence of segmental contribution in cervical spine. Even though the setup of this study was similar, we found less consistent motion patterns in the lumbar spine. We believe several variables between the cervical spine and lumbar spine contribute to our differences in consistency in motion patterns. These variables are as follows: facet orientation, intervertebral discs loadings, the spino-pelvic relationship, and muscle recruitment.

Cervical facet joint surfaces between C3 and T1 have a 45 degrees angle to the transverse plane [27]. In the lumbar spine, the superior articular process is medially orientated, and the inferior articular process is laterally orientated in the sagittal plane, with right angles to the transverse plane [28]. These differences in orientation result in less constrained facet joints of the lumbar spine, resulting in a greater freedom of motion, which could explain a less consistent movement compared to the cervical spine [27]. The uncinate process and uncovertebral joints, found from C3 to C7, also provide stability and mobility of the cervical spine by functioning as a guide rail during flexion and extension and limit rotation and bending, resembling a saddle joint [29]. Since these structures are not present in the lumbar spine, it could lead to less consistent motion patterns due to less constraint of the motion segments. Intervertebral discs of the cervical spine and lumbar spine are both wedged shaped with a larger anterior side of the disc compared to the posterior side [30]. In addition, both discs are elliptical shaped, with a larger cross-sectional area of lumbar intervertebral discs than cervical spine [30]. In this study, it is possible that the axial loading of the intervertebral disc is altered by fixation of the pelvis and the seating position. Nachemson et al [27] described a relative increase in intervertebral disc pressure, ascending from supine to standing to sitting position and from neutral position to flexion. Furthermore, forced anteversion or retroversion of the pelvis caused by the fixed position could influence motion patterns of the lumbar spine during flexion and extension. There is no study that compares motion of the lumbar spine in a standing versus sitting position. The pelvis and abdominal structures also led to overprojection in segment L5S1, making it challenging to trace these segments with the computer software. For this reason, ICCs were determined in this study, resulting in an average ICC of all segments mostly above 0.60, except for L5S1. Furthermore, analyses showed that the lower lumbar spine segments, L4L5 and L5S1, showed inconsistent patterns throughout all recordings. When excluding them from analyses, a more consistent pattern from L1 to L4 appeared. In addition to the difficulty due to overprojection at L5S1, the motion of L4L5 and L5S1 is influenced by more variables compared to the upper lumbar spine, leading to less consistency. The lower lumbar segments function as a kinematic transition zone from a highly mobile region (ie, upper lumbar spine) to an immobile sacroiliac region [31]. For this reason, it is also plausible that pathology mostly occurs in lower lumbar segments.

Finally, muscle recruitment differs between the cervical and lumbar spine. In the cervical spine, the range of rotations is mostly influenced by muscle recruitment, except for the end stages of the motion, which are influenced by gravity [27]. In the lumbar spine, rotation is controlled by muscle recruitments throughout the whole motion. Muscle recruitment and strength is affected by age, sex, motivation, pain, as well as muscle and joint physiology and geometry [27]. This means that mostly interindividual motion differences can be explained by differences in muscle recruitment and strength, which plays a larger part in the lumbar spine compared to the cervical spine motion. In addition, the 4 abdominal muscles (ie, rectus abdominis as well as external and internal oblique and transverse abdominal muscle) have a great influence on flexion of the lumbar spine, with an increased muscle recruitment per degree of flexion [27]. This could also be an explanation for the fact that lumbar flexion patterns are less consistent than lumbar extension patterns.

### Strengths and Limitations

There are multiple strengths to this study. First, the intention of this study was to determine motion patterns of L1 to S1, instead of a selection of vertebrae by using a sufficient field of view. However, especially segment L5S1 was difficult to track due to the overprojection of the pelvis and abdominal structures. Additionally, it is possible that L5S1 also had less focus since this segment was placed at the maximum bottom of the field of view. This resulted in a mean ICC below 0.60 for L5S1 and an inconsistent motion pattern throughout the recordings.

**Figure 3:**
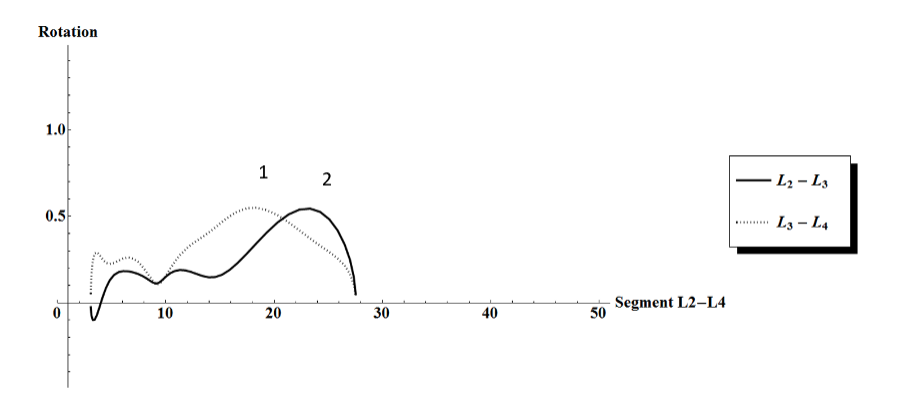
Sagittal rotation in segments in the upper lumbar spine (segments L2 to L4, since L1 fell outside the field of view during extension of the lumbar spine in P1-T1). On the y-axis, the rotation is shown in degrees between successive frames. On the x-axis, cumulative degrees of extension in block L2 to L4 are shown. Peaks of the graphs per segments (L2L3 and L3L4) depict maximum contribution of the segment in a specific phase of the extension. At the end of the extension, the peak of L3L4 was followed by a peak of L2L3. Each series of values undergoes smoothing by means of a low-pass Gaussian digital filter.

Second, this study described motion patterns during maximum flexion and extension of an individual instead of the usually reported fixed ranges to determine physiological motion. Maximum flexion and extension represents the lumbar motion in daily activity better, as it does not limit a person to move within a strict range. Furthermore, patients could move differently because they had to stay within a range of motion, which could influence the muscle recruitment. The downside of using maximum range motion patterns is the possibility of segments moving outside the field of view. This happened once in P1-T1 (Figure 3), resulting in the exclusion of this cinematographic recording from final analyses. However, Figure 2 shows a peak in L3L4, followed by a peak in L2L3 during the last phase of extension, comparable with the abovementioned most consistent sequence of motion.

Finally, since sequence of segmental contribution in the cervical spine showed consistent motion patterns in the study of Boselie et al [18], we used the same imaging technique for recordings, the same computer tracking software, and the same research team in this study [18]. Additionally, cinematographic recordings of all participants were supervised by the same team (IC and CH Christoph) and performed with the use of the same customized chair. The included participants were all male, around the same age, and with a BMI below 25 kg/m^2^ to minimize the influence of age, sex, and body habitus on muscle recruitment and overprojection of abdominal structures. Female participants were excluded to protect their ovaries from direct radiation exposure. However, Staub et al [11], Troke et al [5], Dvorak et al [16], and Wong et al [8] showed no statistically significant difference between sexes in motion of the lumbar spine.

This study also had some limitations. First, sagittal balance parameters were not determined during this study, as femoral heads were not shown in the cinematographic recording. A fixed pelvis could influence the motion of the lumbar spine by forced anteversion or retroversion, which could have been determined using these parameters. Second, the measurement method used to develop the graphics was a time-consuming method. For this reason, the possibility of using artificial intelligence should be investigated to determine if it could lower the workload without losing reliability of the measurements. However, this would be more important for cervical spine analyses, as lumbar spine analyses using this method showed less consistent motion patterns, and therefore, it will have less clinical relevance. It could be possible that another analyzing method should be used to determine physiological motion of the lumbar spine. It has been suggested that center of rotation (COR), defined as the point around which motion segments of the lumbar spine move, could quantify the kinematic features of the lumbar spine [32]. COR of the lumbar spine was the main topic in many previous studies. However, conditions to determine COR varied between studies (eg, symptomatic and asymptomatic participants, different motion tasks, as well as before and after surgery). A current systematic review [32] is analyzing and summarizing data of these different studies to determine if COR could be used to define physiological motion of the lumbar spine. Unfortunately, results are not yet available.

Third, this study was conducted with 11 participants, resulting in 22 cinematographic recordings over 2 time points. Despite this small sample size, we believe that expansion of the study group would not have led to more conclusive results, since there were also intraindividual variabilities between the 2 time points besides interindividual variabilities, and previous research showed consistent results with similar group sizes.

### Conclusions

This study aimed to provide physiological motion patterns of the lumbar spine based on the sequence of segmental contribution. A total of 64% (7/11) of the cinematographic recordings of asymptomatic young male participants showed a consistent pattern at both time points during the last phase of extension, with a peak in rotation in L3L4, followed by a peak in L2L3, and finally, in L1L2. Since 36% (4/11) of the cinematographic recordings did not show a consistent pattern, we believe that physiological motion of the lumbar spine is a broad concept, which cannot be stated in a firm definition using this method. Even in healthy participants, multiple factors are responsible for inconsistencies in lumbar spine motion patterns, which can be aggravated in case of lumbar pathology. For this reason and because of the time-consuming method for analysis, we believe the clinical relevance in this form will be limited, and it should not be used as a diagnostic tool to distinguish between physiological and pathological motions.

## Appendix

Multimedia Appendix 1 Graphs flexion of lumbar motion.

Multimedia Appendix 2 Graphs extension L1 to S1.

Multimedia Appendix 3 Graphs of extension cinematographic recordings L1 to L4; T1 and t2.

## Abbreviations

COR: center of rotation
ICC: intraclass correlation coefficient
T: time point

## Data Availability

The data sets generated and analyzed during this study are available from the corresponding author on reasonable request.
