# Supplementary material for: The Variability of Lumbar Sequential Motion Patterns: Observational Study
Source: JMIR Biomed Eng. 2023 Jun 20;8:e41906. doi: 10.2196/41906 (PMC11041474; doi:10.2196/41906)

P1-T1

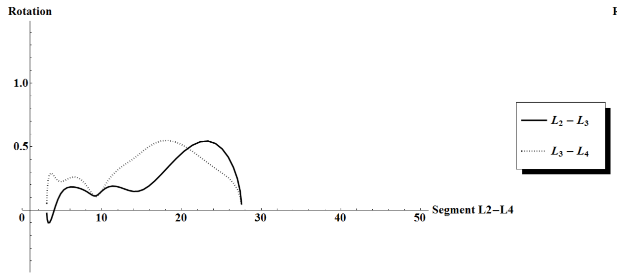

P1-T2

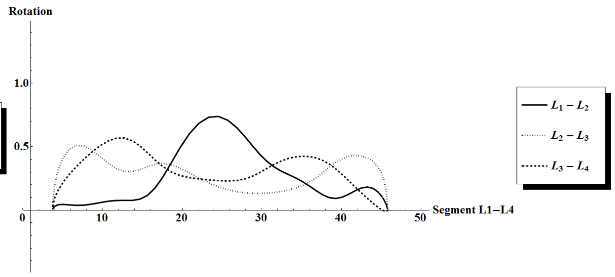

P2-T1

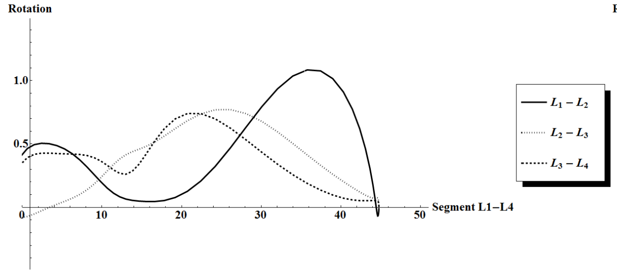

P2-T2

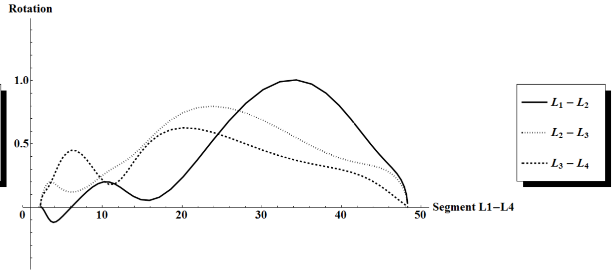

P3-T1

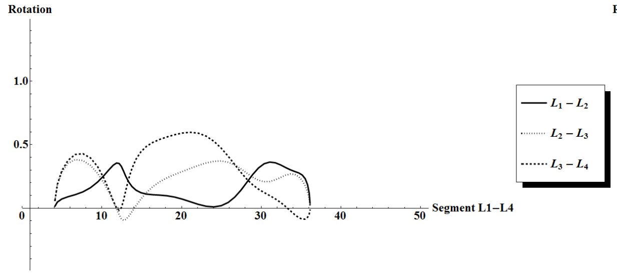

P3-T2

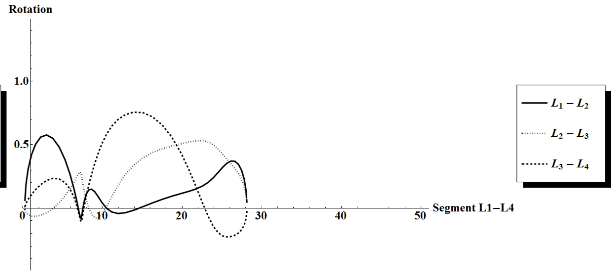

Appendix 3. Extension cinematographic recordings L1 to L4; T1 and T2

P4-T1

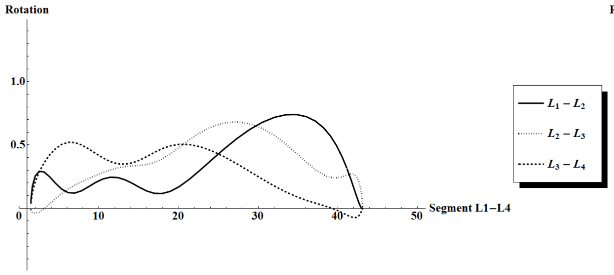

P4-T2

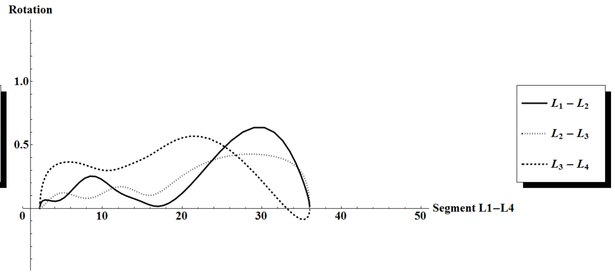

P5-T1

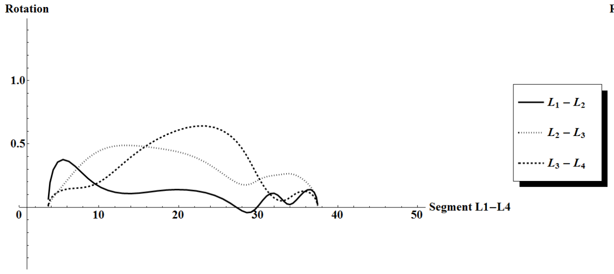

P5-T2

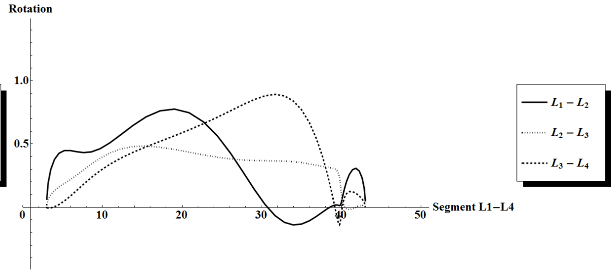

P6-T1

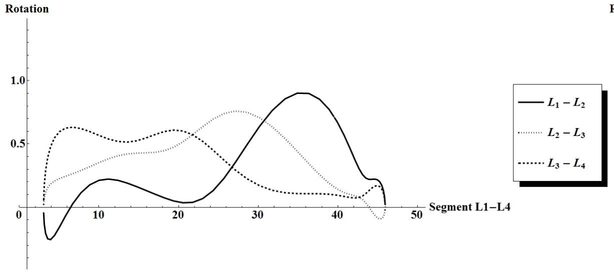

P6-T2

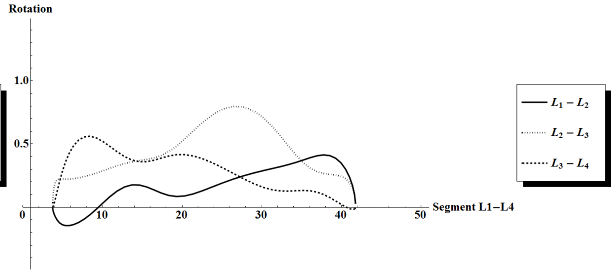

Appendix 3. Extension cinematographic recordings L1 to L4; T1 and T2

P7-T1

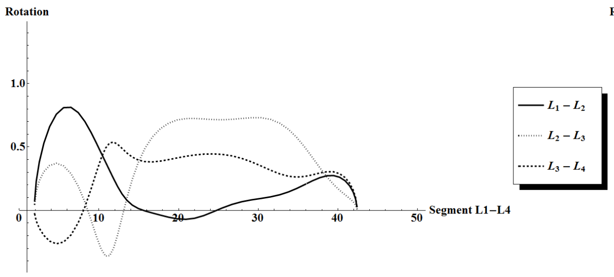

P7-T2

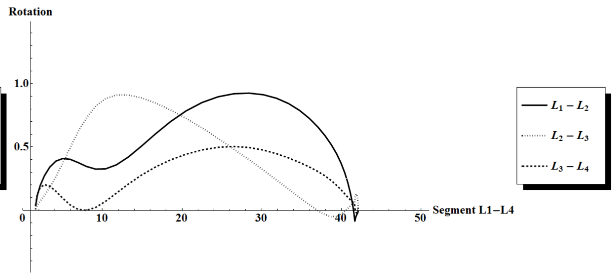

P8-T1

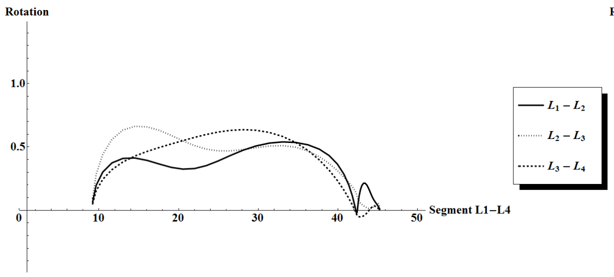

P8-T2

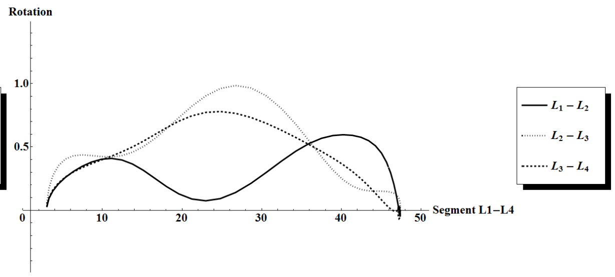

P9-T1

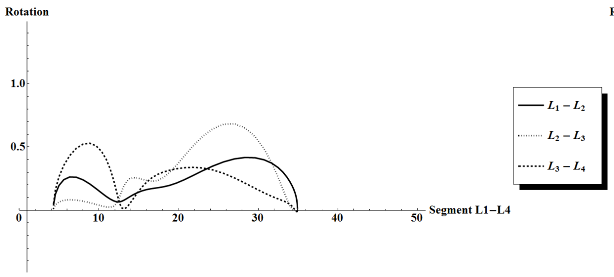

P9-T2

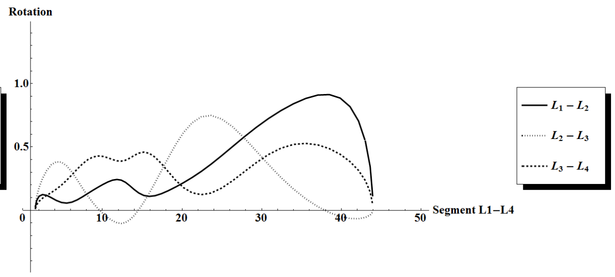

Appendix 3. Extension cinematographic recordings L1 to L4; T1 and T2

P10-T1

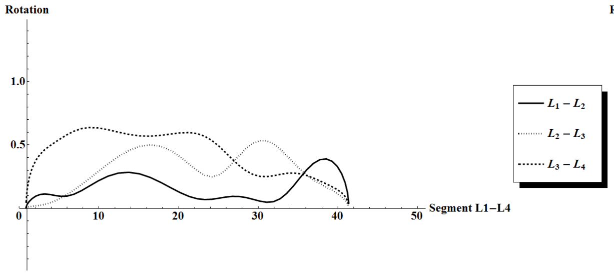

P10-T2

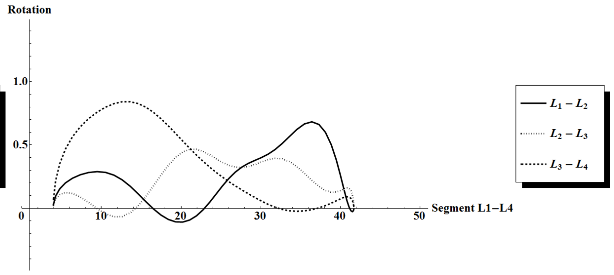

P11-T1

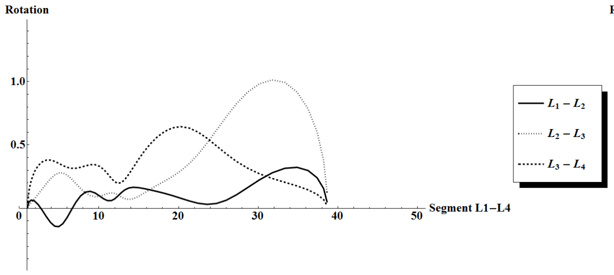

P11-T2

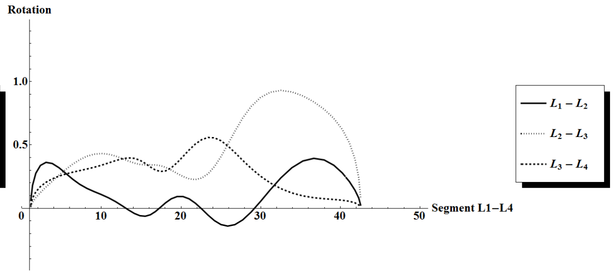

Supplement: Multimedia Appendix 3 [file biomedeng_v8i1e41906_app3.pdf]
